# Efficacy, durability, and safety of faricimab with extended dosing up to every 16 weeks in diabetic macular edema: 2-year results from the Japan subgroup of the phase 3 YOSEMITE trial

**DOI:** 10.1007/s10384-024-01078-y

**Published:** 2024-07-31

**Authors:** Masahiko Shimura, Hideyasu Oh, Tetsuo Ueda, Shigehiko Kitano, Yoshinori Mitamura, Junko Sato, Keisuke Iwasaki, Akito Hirakata, for the YOSEMITE and RHINE Investigators

**Affiliations:** 1 Tokyo Medical University Hachioji Medical Center, 1163, Tatemachi, Hachioji-shi, Tokyo 193-0998 Japan; 2 Hyogo Prefectural Amagasaki General Medical Center, Hyogo, Japan; 3 Nara Medical University Hospital, Nara, Japan; 4 Tokyo Women’s Medical University Hospital, Tokyo, Japan; 5 Tokushima University Graduate School, Tokushima, Japan; 6 Chugai Pharmaceutical Co., Ltd., Tokyo, Japan; 7 Kyorin University School of Medicine, Tokyo, Japan

**Keywords:** Angiopoietin-2, Anti-VEGF therapy, Diabetic macular edema, Faricimab, Vascular stability

## Abstract

**Purpose:**

To evaluate the 2-year efficacy, durability, and safety of faricimab in patients with diabetic macular edema (DME) in the YOSEMITE Japan subgroup.

**Study design:**

YOSEMITE/RHINE (NCT03622580/NCT03622593) subgroup analysis: global, multicenter, randomized, double-masked, active-comparator–controlled, phase 3 faricimab trials.

**Methods:**

Patients were randomized 1:1:1 to intravitreal faricimab 6.0 mg every 8 weeks (Q8W) and per treat-and-extend (T&E) dosing, or aflibercept 2.0 mg Q8W. Outcomes were assessed through year 2 for the YOSEMITE Japan subgroup (N = 60) and the pooled YOSEMITE/RHINE global cohort (N = 1891).

**Results:**

In the YOSEMITE Japan subgroup, 21, 19, and 20 patients were randomized to faricimab Q8W, faricimab T&E, and aflibercept Q8W, respectively (632, 632, and 627 patients in the pooled YOSEMITE/RHINE cohort). Vision gains and anatomic improvements with faricimab at year 1 were maintained over 2 years and were generally consistent between groups. Mean best-corrected visual acuity changes from baseline at year 2 (weeks 92–100 average) for the YOSEMITE Japan subgroup were +12.5, +9.0, and +5.0 letters in the faricimab Q8W, faricimab T&E and aflibercept Q8W arms, respectively (+10.8, +10.4, and +10.3 letters in the pooled YOSEMITE/RHINE cohort). At week 96, 61.1% of the YOSEMITE Japan subgroup and 78.1% of the pooled YOSEMITE/RHINE cohort were on ≥ Q12W dosing. Faricimab was well-tolerated with a safety profile comparable with aflibercept.

**Conclusion:**

Faricimab up to Q16W offered durable vision gains and anatomic improvements up to 2 years in patients with DME in the YOSEMITE Japan subgroup. Outcomes were generally consistent with the pooled YOSEMITE/RHINE cohort.

**Supplementary Information:**

The online version contains supplementary material available at 10.1007/s10384-024-01078-y.

## Introduction

Diabetic macular edema (DME), a consequence of diabetic retinopathy, is a leading cause of visual impairment in Japanese adults [1–3]. In Japan, as is the case globally, intravitreal anti–vascular endothelial growth factor (VEGF) therapy has become the standard of care for patients with center-involving DME with visual impairment [2, 3]. Survey data show that the proportion of ophthalmologists choosing anti-VEGF injections as the first-choice therapy for DME in Japan increased from 73% in 2015 to 81% in 2016–2017 [4, 5].

Real-world studies suggest that the outcomes achieved with anti-VEGF therapies fall short of those anticipated from clinical trials [6, 7]. This is likely in part due to undertreatment associated with the burden of frequent monitoring visits and injections. Data from a large claims database study in Japan indicate that the number of anti-VEGF injections in patients with DME within 1 year after the first injection was 3.5 in 2020 [8] compared with more than 8 in clinical trials [9]. This highlights an unmet need for novel therapies that can alleviate the treatment burden. There is also considerable heterogeneity in response to anti-VEGF treatment in real-world patient populations [10].

Based on the premise that multitargeted treatment strategies for DME may promote vascular stability and durable efficacy beyond those offered by VEGF inhibition alone, faricimab was developed as the first bispecific antibody for intraocular use [11]. Faricimab independently binds and neutralizes both VEGF-A and angiopoietin (Ang)-2, which are involved in regulation of retinal blood vessel growth and integrity, and are upregulated in DME [11, 12]. Faricimab has been approved in multiple countries for the treatment of patients with DME, based on positive 1-year data from the pivotal YOSEMITE and RHINE trials, which are collectively the largest registrational DME program conducted to date [13]. These randomized active-controlled trials of faricimab in patients with DME demonstrate that faricimab 6.0 mg dosed every 8 weeks (Q8W), or according to a personalized treat-and-extend (T&E) regimen, offered noninferior vision gains and anatomic improvements at 1 year compared with aflibercept 2.0 mg Q8W; the primary efficacy endpoint of YOSEMITE and RHINE was change in best-corrected visual acuity (BCVA) from baseline at 1 year, averaged over weeks 48, 52, and 56 [13]. The strong durability of faricimab was evident in the faricimab T&E arm, with > 50% of patients receiving every 16 week (Q16W) dosing at week 52 and > 70% of patients receiving every 12 week (Q12W) dosing or longer [13]. The safety and tolerability profile of faricimab was comparable with aflibercept [13]. Robust vision gains and anatomic improvements achieved with faricimab were maintained up to 2 years with up to Q16W dosing in the faricimab T&E arms [9].

In an analysis of the YOSEMITE Japan subgroup, the efficacy, durability, and safety of faricimab at year 1 were generally consistent with the global results from the pooled YOSEMITE/RHINE cohort [14]. Now that YOSEMITE and RHINE have completed their 96-week treatment periods, we present data from the 2-year analysis of the YOSEMITE Japan subgroup along with an assessment of consistency with the pooled YOSEMITE/RHINE cohort through study end.

## Materials and methods

### Study design

The study design and rationale for YOSEMITE (NCT03622580) and RHINE (NCT03622593) have been previously described [13, 15]. Briefly, YOSEMITE and RHINE were identically designed, global, multicenter, randomized, double-masked, active-comparator–controlled, phase 3 trials of faricimab in patients with DME. Both trials were conducted in accordance with the International Council for Harmonization E6 Guidelines for Good Clinical Practice, the tenets of the Declaration of Helsinki, applicable US Food and Drug Administration regulations, the European Union Clinical Trials Directive (2001/20/EC), and relevant local, state, and federal laws. Study protocols were approved by institutional review boards and ethics committees as applicable, and all patients provided written informed consent.

Patients eligible for inclusion were ≥ 18 years of age and had center-involving DME, defined as BCVA 25–73 Early Treatment Diabetic Retinopathy Study (ETDRS) letters (approximate Snellen equivalent, 20/320–20/40), and central subfield thickness (CST) of ≥ 325 µm for Spectralis spectral domain-optical coherence tomography (SD-OCT) or ≥ 315 µm for Cirrus SD-OCT or Topcon SD-OCT (measured as the average thickness between the internal limiting membrane and Bruch’s membrane in the central 1-mm diameter of the ETDRS grid).

### Treatment protocol

As previously described [13], patients were randomized 1:1:1 to receive intravitreal faricimab 6.0 mg Q8W after 6 initial every-4-week (Q4W) doses, intravitreal faricimab 6.0 mg T&E with up to Q16W dosing intervals after ≥ 4 initial Q4W doses, or intravitreal aflibercept Q8W after 5 initial Q4W doses.

The T&E regimen was a personalized treat-and-extend–based dosing regimen that allowed adjustable dosing up to Q16W [13, 15]. Patients randomized to the faricimab T&E arm initially received faricimab at Q4W intervals. Following first achievement of CST < 325 µm at or after week 12, patients switched to adjustable dosing whereby dosing intervals were extended by 4 weeks (up to Q16W), maintained, or reduced by 4 or 8 weeks (down to Q4W) based on prespecified CST and BCVA criteria at active dosing visits.

To maintain masking, all patients attended Q4W study visits where they received active treatment or sham up to week 96, with a final study visit at week 100.

### Outcome measures

Outcomes were evaluated for Japanese patients enrolled from Japanese study sites in YOSEMITE (no Japanese patients or study sites were included in RHINE) and the pooled YOSEMITE/RHINE trial population. Two-year trial outcomes reported herein were consistent with prespecified endpoints in the primary analysis [13], and included changes in BCVA from baseline at 2 years (defined as the average of weeks 92, 96, and 100) and over time; the proportion of patients in the faricimab T&E dosing arms on Q4W, Q8W, Q12W, or Q16W dosing intervals at week 96 and over time; change in CST from baseline at 2 years and over time; the proportion of patients with absence of DME (CST < 325 μm based on protocol-defined DME) over time; the proportion of patients with absence of intraretinal fluid (IRF) and subretinal fluid (SRF) over time (measured in the central 1 mm ETDRS circle); the proportion of patients with ≥ 2-step improvement on the ETDRS Diabetic Retinopathy Severity Scale (DRSS) at week 96; and the incidence and severity of ocular and nonocular adverse events (AEs) through study end.

### Statistical analysis

Japan subgroup analyses were prespecified in the YOSEMITE trial protocol and efficacy and safety analyses performed were consistent with the primary analysis [13]. Efficacy analyses were based on the intent-to-treat population, grouped by treatment arm at randomization. Adjusted means for continuous endpoints were assessed using a mixed model for repeated measures, adjusted for treatment group, visit, visit-by-treatment group interaction, baseline BCVA or CST (continuous) as applicable, and randomization factors of baseline BCVA and previous intravitreal anti-VEGF therapy. For binary secondary endpoints, weighted proportions were estimated using the Cochran-Mantel-Haenszel method stratified by baseline BCVA score and prior intravitreal anti-VEGF therapy.

Efficacy outcomes for the YOSEMITE Japan subgroup are reported with 95.04% confidence intervals (CIs) to adjust for interim safety assessments conducted through to the completion of the primary analysis [13]. Analyses were conducted by an independent data monitoring committee. Efficacy analyses for the pooled YOSEMITE/RHINE cohort were additionally adjusted or stratified by geographic region and study (YOSEMITE versus RHINE) and 95% CIs are reported. No formal statistical comparisons were made between the YOSEMITE Japan subgroup and pooled YOSEMITE/RHINE cohort; results should be interpreted as exploratory and descriptive.

For all efficacy analyses, intercurrent events due to the COVID-19 pandemic were handled using a hypothetical strategy where all values were censored after the intercurrent event, and intercurrent events not due to COVID-19 were handled using a treatment policy strategy where all observed values were used regardless of the intercurrent event. The robustness of these assumptions and the primary results were demonstrated using sensitivity and supplemental analyses as previously described [13]. Statistical analyses were performed using SAS version 9.4 (SAS Institute, Inc.).

Safety and tolerability were assessed through descriptive summaries of ocular and systemic AEs (coded using Medical Dictionary for Regulatory Activities thesaurus terms), deaths, and ocular assessments through study end.

## Results

### Patient disposition

As reported previously, 1891 patients with DME were enrolled in YOSEMITE (N = 940) and RHINE (N = 951) [13]. This includes patients from the YOSEMITE Japan subgroup. Among the pooled YOSEMITE/RHINE cohort, 632, 632, and 627 patients were randomized to faricimab Q8W, faricimab T&E up to Q16W, and aflibercept Q8W, respectively [13].

The YOSEMITE Japan subgroup included 60 patients enrolled from 27 study sites in Japan (Fig. S1, Online Resource 1) [14]. Of these patients, 21, 19, and 20 were randomized to faricimab Q8W, faricimab T&E up to Q16W, and aflibercept Q8W, respectively [14]. The number of patients who discontinued study treatment and the reasons for discontinuation were generally similar across treatment arms.

### Baseline characteristics

Baseline characteristics for the YOSEMITE Japan subgroup have been previously reported, and were generally well balanced across treatment arms and consistent with the pooled YOSEMITE/RHINE cohort [14].

### Vision outcomes

**Fig. 1:**
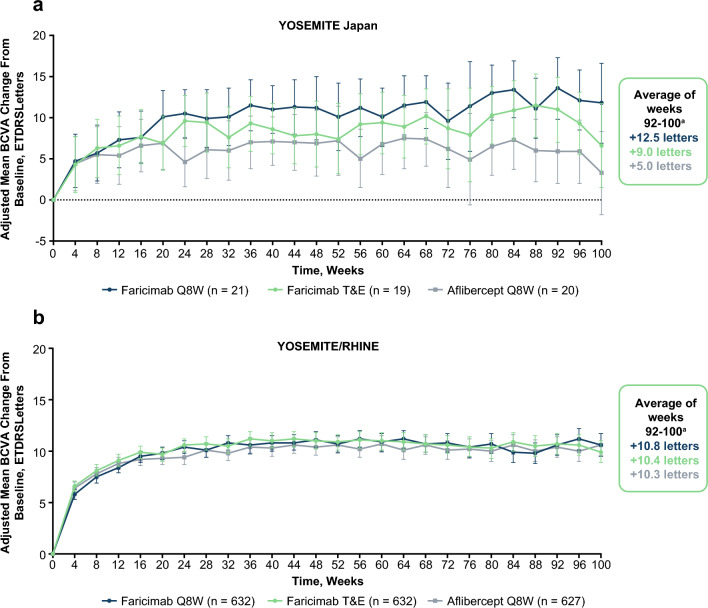
Adjusted mean BCVA change from baseline (ETDRS Letters) in the YOSEMITE Japan subgroup (**a**) and the pooled YOSEMITE/RHINE cohort (**b**). Pooled global cohort includes patients in Japan who were enrolled during the global phase of YOSEMITE/RHINE. Data originally published in Wong et al [9]. ^a^Adjusted mean change from baseline at 2 years, averaged over weeks 92, 96, and 100. Results are based on a mixed model for repeated measures analysis; 95% CI error bars are shown. *BCVA* best-corrected visual acuity, *CI* confidence interval, *ETDRS* Early Treatment Diabetic Retinopathy Study, *Q8W* every 8 weeks, *T&E* treat-and-extend

Vision gains achieved with faricimab at year 1 were maintained over 2 years in the YOSEMITE Japan subgroup and the pooled YOSEMITE/RHINE cohort (Fig. 1). The magnitude of the adjusted mean BCVA change from baseline at year 2 in the faricimab arms of the YOSEMITE Japan subgroup was consistent with the pooled YOSEMITE/RHINE cohort. There was variability in the mean change from baseline between weeks 92 and 100 between treatment arms in the Japan subgroup with adjusted mean 2-year BCVA (95.04% CI) gains of +12.5 (8.8-16.2), +9.0 (5.1-12.8), and +5.0 (1.2-8.8) letters in the faricimab Q8W, faricimab T&E, and aflibercept Q8W arms, respectively. Corresponding differences in adjusted means (95.04% CIs) for faricimab Q8W and faricimab T&E versus aflibercept Q8W were +7.5 (2.2-12.7) and +3.9 (−1.4 to 9.3) letters, respectively. In the pooled YOSEMITE/RHINE cohort, adjusted mean BCVA change (95% CIs) from baseline at year 2 were consistent between the faricimab Q8W (+10.8 [9.8-11.8] letters), faricimab T&E (+10.4 [9.4-11.4] letters), and aflibercept Q8W arms (+10.3 [9.3-11.3] letters) [9]. Corresponding differences in adjusted means (95% CI) for faricimab Q8W and faricimab T&E versus aflibercept Q8W were +0.5 (−0.9 to 1.8) and +0.1 (−1.3 to 1.5) letters, respectively. In both cohorts, 95% CIs were overlapping between treatment arms [9].

In the safety analysis population for the YOSEMITE Japan subgroup, the median (mean [standard deviation]) number of study drug injections over the entire study was 11 (13.2 [3.90]) in the faricimab T&E arm compared with 15 (14.5 [2.40]) in the faricimab Q8W and 15 (14.1 [2.02]) in the aflibercept Q8W arms. During year 2, the median number of study drug injections was 5.0 (5.0 [0.00]), 4.0 (4.4 [2.20]), and 5.0 (4.7 [0.67]) injections in the faricimab Q8W, faricimab T&E, and aflibercept Q8W arms, respectively. Dose interruptions during year 2 were experienced by 1 patient in the faricimab Q8W arm, 2 patients in the faricimab T&E arm, and 1 patient in the aflibercept Q8W arm.

In the safety analysis population for the pooled YOSEMITE/RHINE cohort, the median (mean [standard deviation]) number of study drug injections over the entire study was 11 (11.8 [4.06]) in the faricimab T&E arm compared with 15 (13.5 [2.87]) in the faricimab Q8W and 14 (13.4 [2.70]) in the aflibercept Q8W arms. During year 2, the median (mean [standard deviation]) number of study drug injections were 5.0 (4.7 [0.78]), 3.0 (3.5 [1.88]), and 5.0 (4.5 [0.96]) injections in the faricimab Q8W, faricimab T&E, and aflibercept Q8W arms, respectively. Dose interruptions during year 2 were experienced by 41 patients in the faricimab Q8W arm, 49 patients in the faricimab T&E arm, and 40 patients in the aflibercept Q8W arm.

### Durability outcomes

**Fig. 2:**
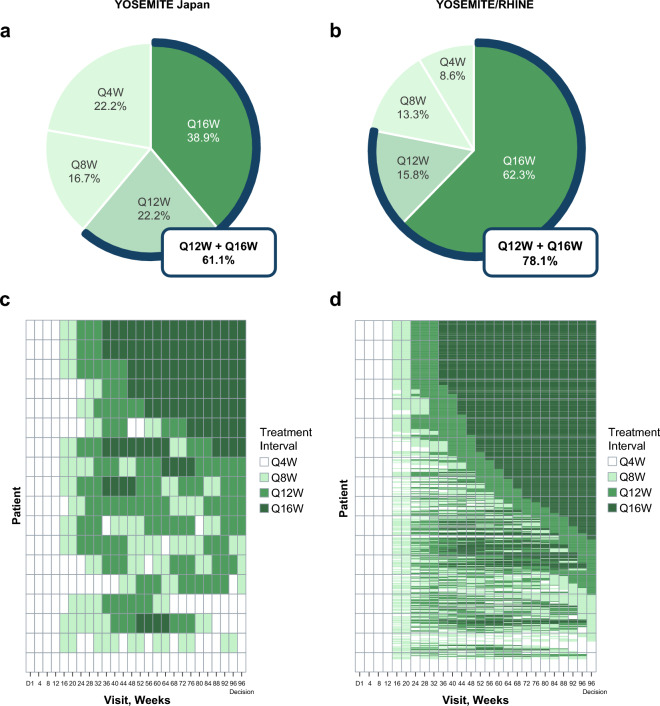
Proportion of patients in the faricimab T&E arm who achieved Q4W, Q8W, Q12W, or Q16W dosing at week 96, and dosing intervals in the faricimab T&E arm through week 96 in the YOSEMITE Japan subgroup (**a**, **c**) and the pooled YOSEMITE/RHINE cohort (**b**, **d**). Pooled global cohort includes patients in Japan who were enrolled during the global phase of YOSEMITE/RHINE. Data originally published in Wong et al [9]. Proportion of patients in the faricimab T&E arm on Q4W, Q8W, Q12W, or Q16W dosing at week 96, among those who had not discontinued the study at the week 96 visit. Treatment interval at week 96 was defined as the treatment interval decision made at that visit. *Q4W* every 4 weeks, *Q8W* every 8 weeks, *Q12W* every 12 weeks, *Q16W* every 16 weeks, *T&E* treat-and-extend

In the YOSEMITE Japan subgroup and the pooled YOSEMITE/RHINE cohort, durable vision gains with faricimab were achieved with T&E dosing up to Q16W (Fig. 2a,b). At week 96, 38.9% of the YOSEMITE Japan subgroup were on Q16W dosing and 61.1% were on Q12W dosing or longer; the corresponding proportions in the pooled YOSEMITE/RHINE cohort were 62.3% and 78.1%, respectively [9].

Faricimab T&E dosing patterns were generally similar between the YOSEMITE Japan subgroup and the pooled YOSEMITE/RHINE cohort (Fig. 2c, d). In the YOSEMITE Japan subgroup and the pooled YOSEMITE/RHINE cohort, 46.2% and 79.3% of patients who achieved ≥ Q12W dosing at week 52 maintained ≥ Q12W dosing through week 96. A similarly high proportion of patients in the YOSEMITE Japan subgroup (71.4%) and the pooled YOSEMITE/RHINE cohort (75.9%) maintained Q16W dosing following achievement at week 52. Q16W dosing was achieved by week 32 (the first time point Q16W dosing was permitted in the study) and maintained through week 96 in 11.1% and 18.3% of patients in the YOSEMITE Japan subgroup and the pooled YOSEMITE/RHINE cohort, respectively. In the YOSEMITE Japan subgroup, 5.6% of patients who extended to Q8W dosing from week 12 remained on Q8W or Q4W dosing and 5.6% of patients never extended beyond Q4W dosing. Corresponding proportions in the pooled YOSEMITE/RHINE cohort were 4.7% and 3.9%, respectively.

### Anatomic outcomes

**Fig. 3:**
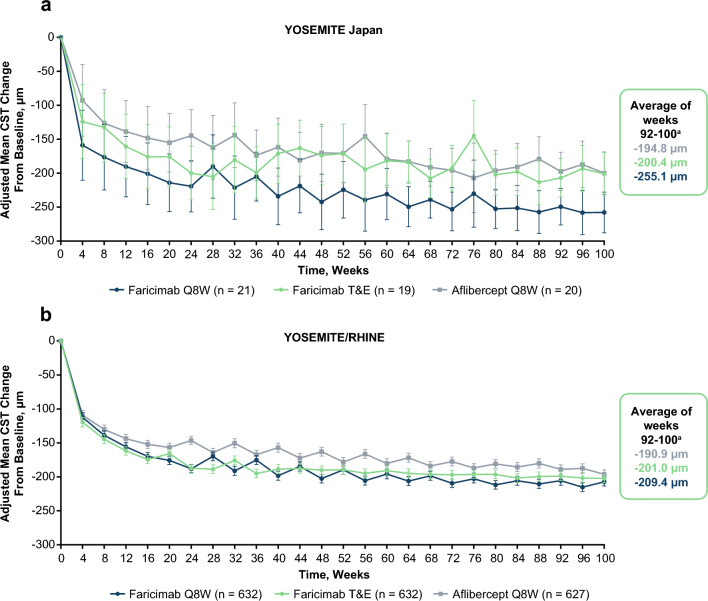
Adjusted mean CST change from baseline in the YOSEMITE Japan subgroup (**a**) and the pooled YOSEMITE/RHINE cohort (**b**). Pooled global cohort includes patients in Japan who were enrolled during the global phase of YOSEMITE/RHINE. Data originally published in Wong et al [9]. ^a^Adjusted mean CST change from baseline at 2 years, averaged over weeks 92, 96, and 100. Results are based on a mixed model for repeated measures analysis; 95% CI error bars are shown. *CI* confidence interval, *CST* central subfield thickness, *Q8W* every 8 weeks, *T&E* treat-and-extend

In the YOSEMITE Japan subgroup, CST reductions achieved with faricimab at year 1 were maintained over 2 years and the mean CST change numerically favored faricimab Q8W over faricimab T&E and aflibercept Q8W at 2 years (Fig. 3). The mean CST changes from baseline across weeks 92–100 were –255.1 µm, –200.4 µm, and –194.8 µm in the faricimab Q8W, faricimab T&E, and aflibercept Q8W arms, respectively. In the pooled YOSEMITE/RHINE cohort, mean reductions in CST at 1 year consistently favored faricimab over aflibercept and these reductions in CST were maintained through year 2. The mean CST changes from baseline across weeks 92–100 were –209.4 µm, –201.0 µm, and –190.9 µm in the faricimab Q8W, faricimab T&E, and aflibercept Q8W arms, respectively [9].

**Fig. 4:**
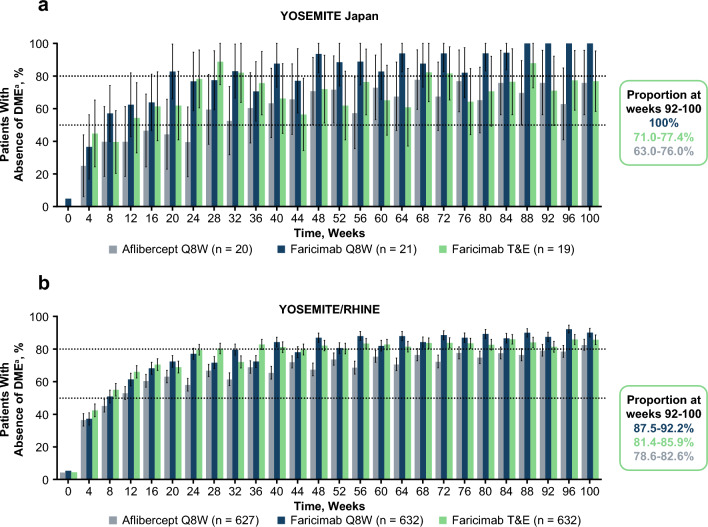
Proportions of patients achieving absence of DME^a^ in the YOSEMITE Japan subgroup (**a**) and the pooled YOSEMITE/RHINE cohort (**b**). Pooled global cohort includes patients in Japan who were enrolled during the global phase of YOSEMITE/RHINE. Data originally published in Wong et al [9]. ^a^Absence of DME was defined as CST < 325 μm, measured as the distance from the internal limiting membrane to Bruch’s membrane. Weighted proportions were estimated using the CMH method, stratified by baseline BCVA (< 64 letters versus ≥ 64 letters), prior intravitreal anti-VEGF therapy (yes versus no) in the YOSEMITE Japan subgroup and the pooled YOSEMITE/RHINE cohort, and by region (United States and Canada versus rest of the world) and study (YOSEMITE versus RHINE) in the pooled YOSEMITE/RHINE cohort. Baseline values are not weighted; 0% of patients had absence of DME at screening, which was up to 28 days ahead of baseline. Weighted proportion for the aflibercept Q8W arm presented for the faricimab Q8W versus aflibercept Q8W comparison. 95% CI error bars are shown; estimates < 0% or > 100% are imputed as 0% or 100%, respectively. *BCVA* best-corrected visual acuity, *CI* confidence interval, *CMH* Cochran-Mantel-Haenszel, *CST* central subfield thickness, *DME* diabetic macular edema, *Q8W* every 8 weeks, *T&E* treat-and-extend, *VEGF* vascular endothelial growth factor

In the YOSEMITE Japan subgroup and the pooled YOSEMITE/RHINE cohort, the proportion of faricimab-treated patients achieving absence of DME was maintained or improved from year 1 through year 2 (Fig. 4). Numerically more patients in the YOSEMITE Japan subgroup and the pooled YOSEMITE/RHINE cohort achieved absence of DME (defined as CST < 325 μm) in the faricimab arms compared with the aflibercept arm at year 2. The proportion of patients in the YOSEMITE Japan subgroup achieving absence of DME across weeks 92–100 was 100%, 71.0–77.4%, and 63.0–76.0% in the faricimab Q8W, faricimab T&E, and aflibercept Q8W arms, respectively. In the pooled YOSEMITE/RHINE cohort, the proportion of patients achieving absence of DME across weeks 92–100 was 87.5–92.2%, 81.4–85.9%, and 78.6–82.6% in the faricimab Q8W, faricimab T&E, and aflibercept Q8W arms, respectively [9].

A similar pattern was observed for absence of IRF in the YOSEMITE Japan subgroup and the pooled YOSEMITE/RHINE cohort, except that the proportion of patients was numerically higher in the faricimab arms than the aflibercept Q8W arm in the YOSEMITE Japan subgroup (Fig. S2a, b, Online Resource 1). In the YOSEMITE Japan subgroup, the proportion of patients with absence of IRF was numerically reduced in the faricimab T&E arm at year 2 versus year 1. The proportions of patients achieving absence of IRF across weeks 92–100 were 55.7–70.8%, 17.3–29.0%, and 37.9–56.0% in the faricimab Q8W, faricimab T&E, and aflibercept Q8W arms, respectively. In the pooled YOSEMITE/RHINE cohort, the proportion of patients with absence of IRF was generally numerically higher in the faricimab Q8W and T&E arms than the aflibercept Q8W arms. The proportions of patients achieving absence of IRF across weeks 92–100 were 57.2–62.7%, 44.1–48.4%, and 36.2–41.3% in the faricimab Q8W, faricimab T&E, and aflibercept Q8W arms, respectively [9].

Absence of SRF through week 100 was high and generally consistent across treatment arms and across the YOSEMITE Japan subgroup and the pooled YOSEMITE/RHINE cohort; 58–76% of patients in the YOSEMITE Japan subgroup and 62–66% of patients in the pooled YOSEMITE/RHINE cohort had absence of SRF at baseline, which increased to near 100% for all groups by week 16 and was maintained through week 100 (Fig. S2c, d, Online Resource 1).

### DRSS improvement

**Fig. 5:**
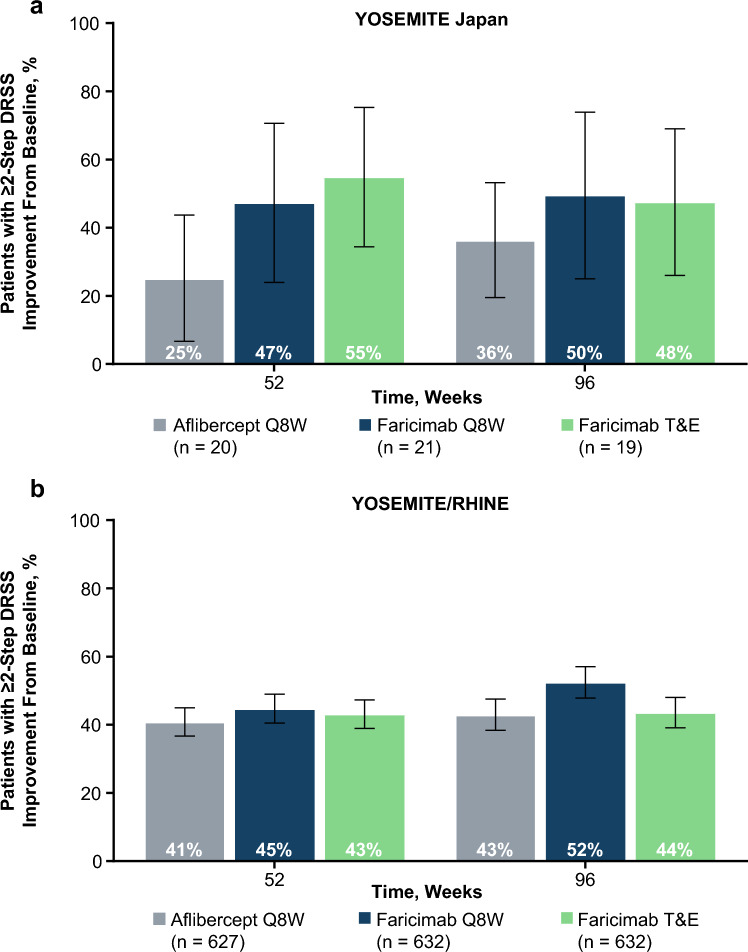
Proportion of patients with ≥ 2-Step DRSS improvement in the YOSEMITE Japan subgroup (**a**) and the pooled YOSEMITE/RHINE cohort (**b**). Pooled global cohort includes patients in Japan who were enrolled during the global phase of YOSEMITE/RHINE. Data originally published in Wong et al [9]. Analyses included patients with evaluable color fundus photograph images at baseline and week 52 and/or week 96. Weighted proportions were estimated using the CMH method, stratified by baseline BCVA (< 64 letters versus ≥ 64 letters), prior intravitreal anti-VEGF therapy (yes versus no) in the YOSEMITE Japan subgroup and the pooled YOSEMITE/RHINE cohort, and by region (United States and Canada versus rest of the world) and study (YOSEMITE versus RHINE) in the pooled YOSEMITE/RHINE cohort. Weighted proportions for the aflibercept Q8W arm presented for the faricimab Q8W versus aflibercept Q8W comparison. 97.52% CI error bars are shown at week 52; 95.04% CI error bars are shown at week 96. *BCVA* best-corrected visual acuity, *CI* confidence interval, *CMH* Cochran-Mantel-Haenszel, *DRSS* Diabetic Retinopathy Severity Scale, *ETDRS* Early Treatment Diabetic Retinopathy Study, *Q8W* every 8 weeks; *T&E* treat-and-extend, *VEGF* vascular endothelial growth factor

Rates of at least 2-step DRSS improvement were largely consistent across treatment arms and between the YOSEMITE Japan subgroup and the pooled YOSEMITE/RHINE cohort at weeks 52 and 96, although there was a numerical trend for higher proportions in the faricimab arms versus the aflibercept arm in the YOSEMITE Japan subgroup (Fig. 5). The proportion of patients achieving at least 2-step DRSS improvement at week 96 was 50%, 48%, and 36% in the YOSEMITE Japan subgroup in the faricimab Q8W, faricimab T&E, and aflibercept Q8W arms, respectively, and 52%, 44%, and 43% in the pooled YOSEMITE/RHINE cohort [9].

### Safety outcomes

In the YOSEMITE Japan subgroup and the pooled YOSEMITE/RHINE cohort, faricimab was well tolerated through study end, with a safety profile comparable with aflibercept (Table 1). In the pooled YOSEMITE/RHINE cohort and the YOSEMITE Japan subgroup, the incidence of ocular AEs through study end was comparable between patients receiving faricimab Q8W (49.7% and 42.9%, respectively), faricimab T&E (49.2% and 42.1%), and aflibercept Q8W (45.4% and 50.0%), with most events being mild or moderate in severity. Serious ocular AEs in the pooled cohort and the YOSEMITE Japan subgroup were low and comparable between the faricimab Q8W (4.1% and 4.8%, respectively), faricimab T&E (5.4% and 5.3%), and aflibercept Q8W (3.2% and 10.0%) treatment arms. The incidence of nonocular AEs was also comparable across treatment arms and similar between the YOSEMITE Japan subgroup and the pooled YOSEMITE/RHINE cohort. No Antiplatelet Trialists’ Collaboration events were reported in the YOSEMITE Japan subgroup.

**Table 1:** Summary of key adverse events through week 100 for the YOSEMITE Japan subgroup and the pooled YOSEMITE/RHINE cohort

	YOSEMITE Japan subgroup			Pooled YOSEMITE/RHINE cohort		
	Faricimab Q8W (n = 21)	Faricimab T&E (n = 19)	Aflibercept Q8W (n = 20)	Faricimab Q8W (n = 630)	Faricimab T&E (n = 632)	Aflibercept Q8W (n = 625)
Total number of AEs	92	80	77	3279	3052	2862
Total number of SAEs	4	6	8	407	353	363
Patients with ≥ 1 ocular AE, n (%)^a^	9 (42.9%)	8 (42.1%)	10 (50.0%)	313 (49.7%)	311 (49.2%)	284 (45.4%)
Patients with ≥ 1 ocular SAE, n (%)^a^	1 (4.8%)	1 (5.3%)	2 (10.0%)	26 (4.1%)	34 (5.4%)	20 (3.2%)
Patients with ≥ 1 nonocular AE, n (%)	15 (71.4%)	16 (84.2%)	15 (75.0%)	460 (73.0%)	469 (74.2%)	473 (75.7%)
Patients with ≥ 1 nonocular SAE, n (%)	2 (9.5%)	3 (15.8%)	3 (15.0%)	175 (27.8%)	161 (25.5%)	173 (27.7%)
Patients with ≥ 1 treatment-related ocular AE, n (%)^a^	2 (9.5%)	2 (10.5%)	0	20 (3.2%)	21 (3.3%)	21 (3.4%)
Patients with ≥ 1 treatment-related ocular SAE, n (%)^a^	0	1 (5.3%)	0	0	7 (1.1%)	0
Patients with ≥ 1 ocular AE of special interest, n (%)^b^	1 (4.8%)	1 (5.3%)	1 (5.0%)	25 (4.0%)	33 (5.2%)	20 (3.2%)
Intraocular inflammation events, n (%)^c^	1 (4.8%)	1 (5.3%)	0	9 (1.4%)	11 (1.7%)	7 (1.1%)
Uveitis	1 (4.8%)	1 (5.3%)	0	3 (0.5%)	4 (0.6%)	0
Iritis	0	0	0	1 (0.2%)	4 (0.6%)	2 (0.3%)
Iridocyclitis	0	0	0	2 (0.3%)	3 (0.5%)	1 (0.2%)
Vitritis	0	0	0	2 (0.3%)	0	2 (0.3%)
Post-procedural inflammation	0	0	0	1 (0.2%)	1 (0.2%)	2 (0.3%)
Chorioretinitis	0	0	0	0	1 (0.2%)	0
Keratic precipitates	0	1 (5.3%)	0	0	1 (0.2%)	0
Keratouveitis	0	0	0	0	1 (0.2%)	0
Endophthalmitis events, n (%)	0	0	0	2 (0.3%)	4 (0.6%)	1 (0.2%)
Retinal vasculitis events, n (%)	0	0	0	0	0	0
Retinal occlusive events, n (%)						
Retinal vein occlusion	0	1 (5.3%)	0	1 (0.2%)	4 (0.6%)	0
Retinal artery occlusion	0	0	0	1 (0.2%)	2 (0.3%)	2 (0.3%)
Retinal artery embolism	0	0	0	0	0	1 (0.2%)
Arterial occlusive disease	0	0	0	0	0	1 (0.2%)
APTC events, n (%)^d^	0	0	0	34 (5.4%)	30 (4.7%)	32 (5.1%)

Results are presented for safety-evaluable population. Percentages are based on n values in the column headings; multiple occurrences of the same AE in an individual are counted only once

*AE* adverse event, *APTC* Antiplatelet Trialists’ Collaboration, *BCVA* best-corrected visual acuity, *Q8W* every 8 weeks, *T&E* treat-and-extend

^a^Ocular AEs in the study eye only are presented; ^b^Ocular AEs of special interest were defined as events associated with severe intraocular inflammation, events requiring surgical or medical intervention to prevent permanent loss of sight or events associated with BCVA loss of ≥ 30 letters for > 1 hour; ^c^Excluding endophthalmitis; ^d^APTC events were adjudicated by an external independent committee; all other events were investigator reported. Includes AEs with onset from the first dose of study drug through study end

In the YOSEMITE Japan subgroup and the pooled YOSEMITE/RHINE cohort, the incidence of intraocular inflammation AEs through study end was low across the faricimab Q8W (4.8% [1/21] and 1.4% [9/630], respectively), faricimab T&E (5.3% [1/19] and 1.7% [11/632]), and aflibercept Q8W (0% and 1.1% [7/625]) treatment arms. Noninflammatory retinal occlusive events through study end were low and comparable across treatment arms and trial populations, and no cases of retinal vasculitis or occlusive retinal vasculitis were reported.

## Discussion

Following the completion of the YOSEMITE and RHINE trials and publication of year 2 data [9], the current analysis was performed to extend the previously reported YOSEMITE Japan subgroup year 1 analysis [14] through to study end along with an assessment of consistency with the pooled YOSEMITE/RHINE cohort. Data from the YOSEMITE Japan subgroup demonstrated that vision gains, anatomic improvements, and extended durability with faricimab up to Q16W were maintained through year 2, in line with what was observed in the pooled YOSEMITE/RHINE cohort and the individual YOSEMITE and RHINE trial findings [9].

BCVA gains, anatomic outcomes, and safety of faricimab in the YOSEMITE Japan subgroup were generally consistent with the global YOSEMITE/RHINE results [9]. BCVA gains with faricimab at year 1 were maintained through year 2 and remained comparable with those seen with aflibercept. Improved anatomic outcomes with faricimab versus aflibercept in the YOSEMITE Japan subgroup were maintained over 2 years: numerical advantages in favor of faricimab were observed in the change from baseline in CST and in the proportion of patients achieving absence of DME and IRF.

Durable vision gains and anatomic outcomes with faricimab were achieved in the YOSEMITE Japan subgroup, with more than 60% of patients on Q12W or Q16W dosing at week 96. In a real-world study from 2019, a maximum injection interval of 14 weeks and a mean injection interval of 8.5 ± 2.9 weeks was reported among 75 treatment-naïve eyes with DME treated with anti-VEGF (ranibizumab or aflibercept) according to a T&E schedule [16]. This is lower than the maximum injection interval of 16 weeks achieved in this study. Furthermore, in another retrospective consecutive case study, the mean recurrence interval (time between previous injection and criteria for an additional injection being met) of previous anti-VEGF injection was 5.8 ± 2.5 weeks, which was significantly extended to 10.8 ± 4.9 weeks (p=0.0005) following switching to faricimab [17]. These findings reflect an opportunity for reduced treatment burden with faricimab relative to current options.

Faricimab continued to be well tolerated in the YOSEMITE Japan subgroup through year 2, with a safety profile comparable with that of aflibercept. Additional data regarding the longer-term safety and efficacy of faricimab in DME will come from the RHONE-X extension trial (NCT04432831) for patients completing YOSEMITE or RHINE and the VOYAGER study (NCT05476926), a real-world study of patients with DME or neovascular age-related macular degeneration who are treated with faricimab or the Port Delivery System with ranibizumab in routine clinical practice.

Although general trends were similar between the YOSEMITE Japan subgroup and the pooled YOSEMITE/RHINE cohort [9], some differences were noted, including increased variability between treatment arms in the YOSEMITE Japan subgroup versus the pooled cohort. This was apparent in the adjusted mean BCVA changes from baseline between weeks 92 and 100 and in the proportion of patients with ≥ 2-step DRSS improvement at study end. Additionally, the proportion of patients in the faricimab T&E arm who achieved Q12W or Q16W dosing was lower for the YOSEMITE Japan subgroup compared with the pooled YOSMITE/RHINE cohort. These differences may be a reflection of the smaller number of patients in the YOSEMITE Japan subgroup. There was a trend toward greater improvement in DRSS with faricimab in the pooled YOSEMITE/RHINE cohort [9]. Although a similar trend was observed in the YOSEMITE Japan subgroup, the small number of patients preclude any definite conclusions from being drawn. However, these findings are nevertheless consistent with faricimab improving diabetic retinopathy by inhibiting Ang-2 and stabilizing blood vessels [18]. This is further highlighted in a recently published descriptive review from Japan, which shows a reduction in microaneurysm count in a patient treated with faricimab [19]. Another difference is that the proportions of patients achieving absence of IRF decreased slightly in the faricimab T&E arm during the second year in the YOSEMITE Japan subgroup, but not in the pooled YOSEMITE/RHINE cohort [9]. Of note, there was a high degree of variability and a small overall sample size in the YOSEMITE Japan subgroup, which may have contributed to these differences.

This study was limited by the small sample size of the YOSEMITE Japan subgroup. Findings from the prospective, open-label, multicenter SWAN study (jRCTs031230213) will provide further information on the efficacy and safety of faricimab in a larger cohort of Japanese patients with DME. Additionally, no formal statistical comparisons were made between treatment arms or with the pooled cohort; thus, these findings should be interpreted as exploratory and descriptive.

In conclusion, year 2 data from the YOSEMITE Japan subgroup are generally consistent with the pooled YOSEMITE/RHINE cohort [9], and show that dual Ang-2/VEGF-A inhibition with faricimab offered comparable vision stability and numerically greater fluid control versus aflibercept, while also demonstrating strong durability, with the potential for extended dosing up to every 16 weeks. These results support dual Ang-2/VEGF-A inhibition with faricimab as a treatment for DME that provides durable efficacy, extended treatment intervals, and, consequently, may reduce the treatment burden for patients.

## Supplementary Information

Below is the link to the electronic supplementary material.

Supplementary file1 Patient flow diagram for YOSEMITE Japan subgroup.Q8W every 8 weeks, T&E treat-and-extend (PDF 42 KB)

Supplementary file2 Proportions of patients achieving absence of IRFa or SRF in the YOSEMITE Japan subgroup (a, c) and the pooled YOSEMITE/RHINE cohort (b, d). IRF and SRF were measured in the central 1-mm diameter of the ETDRS grid. Weighted proportions were estimated using the CMH method, stratified by baseline BCVA (< 64 letters versus ≥ 64 letters), prior intravitreal anti-VEGF therapy (yes versus no) in the YOSEMITE Japan subgroup and the pooled YOSEMITE/RHINE cohort, and by region (United States and Canada versus rest of the world) and study (YOSEMITE versus RHINE) in the pooled YOSEMITE/RHINE cohort. Baseline values are not weighted. Weighted proportion for the aflibercept Q8W arm presented for the faricimab Q8W versus aflibercept Q8W comparison. 95% CI error bars are shown; estimates < 0% or > 100% are imputed as 0% or 100%, respectively. BCVA best-corrected visual acuity, CI confidence interval, CMH Cochran-Mantel-Haenszel, ETDRS Early Treatment Diabetic Retinopathy Study, IRF intraretinal fluid, Q8W every 8 weeks, SRF subretinal fluid, T&E treat-and-extend, VEGF vascular endothelial growth factor (PDF 61 KB)

## Data Availability

For eligible studies, qualified researchers may request access to individual patient-level clinical data through a data request platform. At the time of writing, this request platform is Vivli (https://vivli.org/ourmember/roche/). For up-to-date details on Roche’s Global Policy on the Sharing of Clinical Information and how to request access to related clinical study documents, see here (https://go.roche.com/data_sharing). Anonymized records for individual patients across more than 1 data source external to Roche cannot, and should not, be linked due to a potential increase in risk of patient re-identification.

## Declarations

### Meeting presentation

Data reported in this manuscript were presented in part at 127th Annual Meeting of the Japanese Ophthalmological Society; Tokyo, Japan; 6–9 April 2023.
