# Supplementary figures and images for: Efficacy, durability, and safety of faricimab with extended dosing up to every 16 weeks in diabetic macular edema: 2-year results from the Japan subgroup of the phase 3 YOSEMITE trial
Source: Jpn J Ophthalmol. 2024 Jul 31;68(5):511–22. doi: 10.1007/s10384-024-01078-y (PMC11420323; doi:10.1007/s10384-024-01078-y)

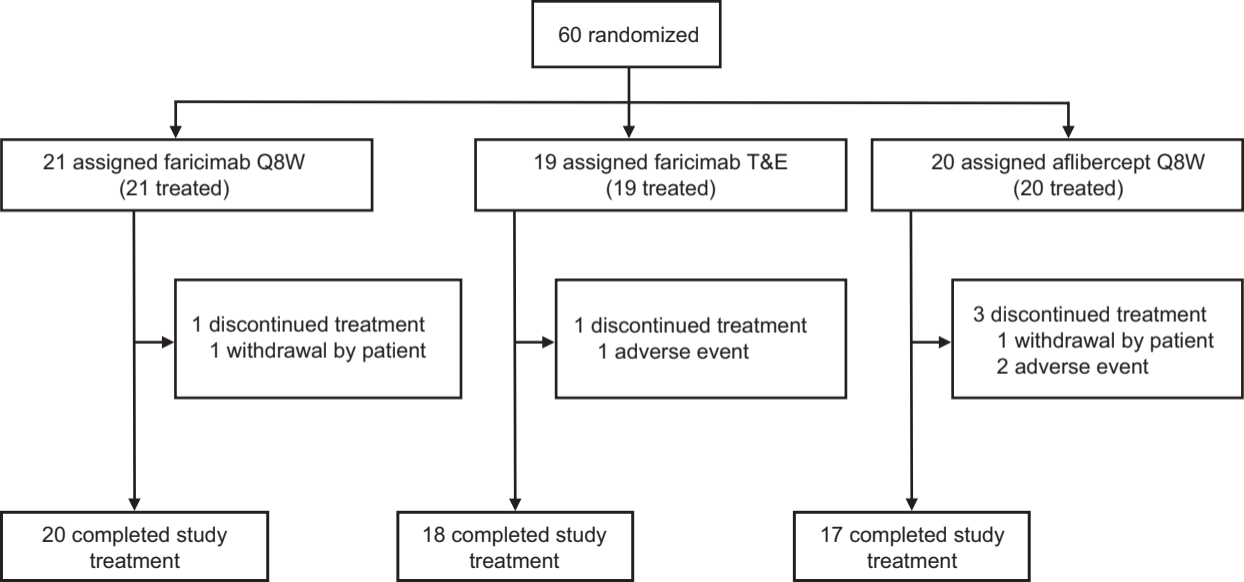

Supplement: Supplementary file 1 — Supplementary file1 Patient flow diagram for YOSEMITE Japan subgroup.Q8W every 8 weeks, T&E treat-and-extend (PDF 42 KB) [file 10384_2024_1078_MOESM1_ESM.pdf]

## YOSEMITE Japan

a

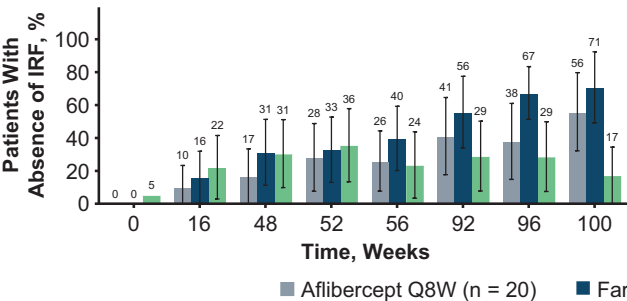

c

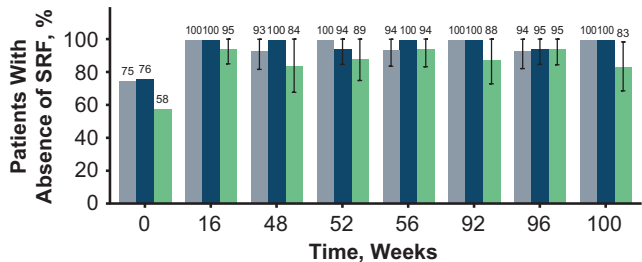

b

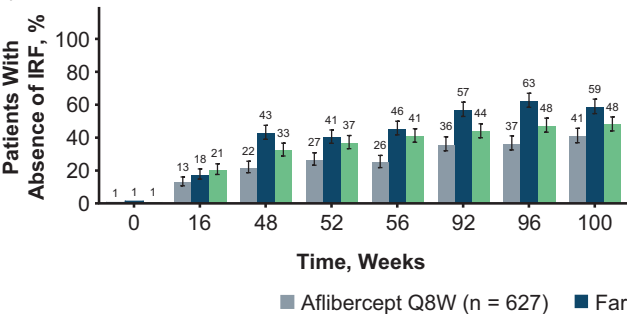

d

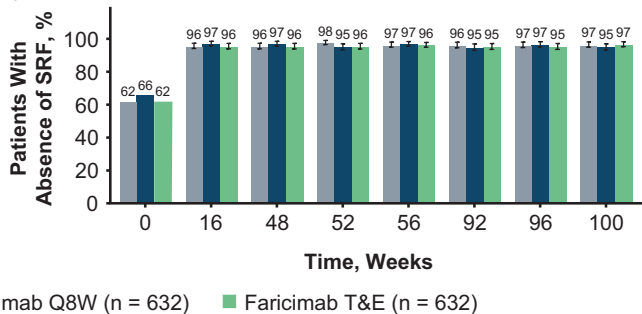

Supplement: Supplementary file 2 — Supplementary file2 Proportions of patients achieving absence of IRFa or SRF in the YOSEMITE Japan subgroup (a, c) and the pooled YOSEMITE/RHINE cohort (b, d). IRF and SRF were measured in the central 1-mm diameter of the ETDRS grid. Weighted proportions were estimated using the CMH method, stratified by baseline BCVA (< 64 letters versus ≥ 64 letters), prior intravitreal anti-VEGF therapy (yes versus no) in the YOSEMITE Japan subgroup and the pooled YOSEMITE/RHINE cohort, and by region (United States and Canada versus rest of the world) and study (YOSEMITE versus RHINE) in the pooled YOSEMITE/RHINE cohort. Baseline values are not weighted. Weighted proportion for the aflibercept Q8W arm presented for the faricimab Q8W versus aflibercept Q8W comparison. 95% CI error bars are shown; estimates < 0% or > 100% are imputed as 0% or 100%, respectively. BCVA best-corrected visual acuity, CI confidence interval, CMH Cochran-Mantel-Haenszel, ETDRS Early Treatment Diabetic Retinopathy Study, IRF intraretinal fluid, Q8W every 8 weeks, SRF subretinal fluid, T&E treat-and-extend, VEGF vascular endothelial growth factor (PDF 61 KB) [file 10384_2024_1078_MOESM2_ESM.pdf]
